# Hydrogen sulfide donor activates AKT-eNOS signaling and promotes lymphatic vessel formation

**DOI:** 10.1371/journal.pone.0292663

**Published:** 2023-10-26

**Authors:** Ravi Varma Aithabathula, Naveed Pervaiz, Ishita Kathuria, Mallory Swanson, Udai P. Singh, Santosh Kumar, Frank Park, Bhupesh Singla

**Affiliations:** Department of Pharmaceutical Sciences, College of Pharmacy, The University of Tennessee Health Science Center, Memphis, TN, United States of America; Longgang Otorhinolaryngology Hospital & Shenzhen Key Laboratory of Otorhinolaryngology, Shenzhen Institute of Otorhinolaryngology, CHINA

## Abstract

The lymphatic network is pivotal for various physiological functions in the human body. Accumulated evidence supports the role of therapeutic lymphangiogenesis in the treatment of several pathologies. Endogenous gasotransmitter, hydrogen sulfide (H_2_S) has been extensively studied for its potential as a pro-angiogenic factor and vascular function modulator. However, the role of H_2_S in governing lymphatic vessel formation, and underlying molecular mechanisms are understudied. The present study was designed to investigate the effects of H_2_S donor sodium hydrogen sulfide (NaHS) on lymphatic vascularization and pro-angiogenic signaling pathways using both in vitro and in vivo approaches. In vitro dose-response experiments showed increased proliferation and tube formation by NaHS-treated human lymphatic endothelial cells (LECs) compared with control cells. Immunoblotting performed with LEC lysates prepared after time-course NaHS treatment demonstrated increased activation of ERK1/2, AKT and eNOS after 20 min of NaHS stimulation. Further, NaHS treatment induced nitric oxide production, reduced reactive oxygen species generation, and promoted cell cycle in LECs. Additional cell cycle analysis showed that NaHS treatment abrogates oxidized LDL-induced cell cycle arrest in LECs. The results of in vivo Matrigel plug assay revealed increased lymphatic vessel density in Matrigel plugs containing NaHS compared with control plugs, however, no significant differences in angiogenesis and immune cell infiltration were observed. Collectively, these findings suggest that H_2_S donor NaHS promotes lymphatic vessel formation both in vitro and in vivo and may be utilized to promote reparative lymphangiogenesis to alleviate lymphatic dysfunction-related disorders.

## Introduction

The lymphatic vasculature, which is constituted by lymphatic vessels (LVs), lymph nodes (LNs) and lymphoid organs, plays an important role in maintaining interstitial fluid homeostasis, adaptive immunity, regulation of inflammatory responses, and host defense via draining extravasated interstitial fluid, immune cells, inflammatory cytokines and antigens from peripheral tissue to the draining LNs and back into the systemic circulation [1–3]. The formation of new LVs from the preexisting lymphatics referred as lymphangiogenesis, is a complex physiological mechanism resulting from the proliferation, migration, sprouting, and formation of vessel-like structures by lymphatic endothelial cells (LECs) [4]. Lymphangiogenesis in adults is known to exert both detrimental and beneficial effects [5]. LVs serve as a route for tumor cells to egress from the tumor tissue and spread to distant organs suggesting the role of LVs in tumor metastasis [6]. On the other hand, LVs aid in the clearance of excess interstitial fluids and proinflammatory cells and mediators from the site of inflammation, thereby causing the resolution of inflammation. In recent years, therapeutic lymphangiogenesis has gained significant attention for the treatment of various metabolic, renal and cardiovascular diseases [5, 7]. However, our knowledge about the inhibitors and stimulators of lymphangiogenesis in adults is still limited.

Hydrogen sulfide (H_2_S), an endogenous gaseous molecule is present in minute quantities within the body. Like other gaseous molecules including nitric oxide (NO) and carbon monoxide, H_2_S plays both physiological and pathological roles. Treatment with H_2_S donors has been shown to promote vasorelaxation, stimulate angiogenesis and serve therapeutic benefits in various disease models including cardiac ischemia-reperfusion injury [8], pressure overload-induced heart failure [9], high fat diet-induced cardiac dysfunction, [10–13]. A recent study by Suzuki et al. reported the beneficial effects of diallyl trisulfide (H_2_S donor) treatment on lymphatic vessel growth and tail lymphedema [14]. However, the molecular mechanisms by which H_2_S donor stimulates lymphatic vascularization are largely unknown. Therefore, the present study was designed to investigate the effects of H_2_S donor (sodium hydrogen sulfide, NaHS) on lymphatic vessel formation, pro-angiogenic pathway (AKT-eNOS), NO production and cell cycle progression. Our findings demonstrate that NaHS treatment induces lymphatic vessel formation, promotes NO production, and prevents oxidized LDL (oxLDL)-induced inhibition of cell cycle in LECs.

## Materials and methods

### Reagents and antibodies

Growth factor-reduced Matrigel matrix was purchased from Corning (Bedford, MA, USA). FxCycle™ PI/RNase Staining Solution, H2DCFDA, DAF-FM diacetate, and DAPI were bought from Life Technologies Corporation (Eugene, OR, USA). Anhydrous sodium sulfide (Na_2_S), Pierce BCA protein assay kit, RIPA buffer, protease and phosphatase inhibitor mini tablets, RevertAid RT Reverse Transcription Kit, PowerUp SYBR™ Green Master Mix, TRIzol Reagent and CD68 antibody were procured from Thermo Scientific (Rockford, IL, USA). Na_2_S solution was prepared in molecular biology grade water. Human oxidized low-density lipoprotein (oxLDL) was acquired from Kalen Biomedical, LLC (Montgomery, MD, USA). Cell proliferation reagent WST-1 was obtained from Roche Diagnostics GmbH (Mannheim, Germany). Phospho-eNOS (Ser1177), phospho-AKT (Ser473), phospho-ERK1/2 (Thr202/Tyr204), total AKT, total ERK1/2, total eNOS, Ki67, and CD45 antibodies were purchased from Cell Signaling Technology (Danvers, MA, USA). GAPDH antibody was procured from Santa Cruz Biotechnology (Dallas, TX, USA). LYVE-1 antibody was purchased from Abcam (Cambridge, MA, USA). CD31 antibody was obtained from R&D Systems, Inc. (Minneapolis, MN, USA). Ready-to-use intercept blocking buffer to block the nitrocellulose membranes was purchased from Li-Cor Biosciences (Lincoln, NE, USA).

### Animals

Ten-week-old male C57BL/6 (wild-type, JAX, stock # 000664) mice were used in the present study. All mice were kept in accordance with the Institutional guidelines, fed a standard chow diet, and maintained under a 12 h light/12 h dark cycle with ad libitum access to food and drinking water. All animal procedures were performed after getting approval (protocol # 22–0319) from the University of Tennessee Health Science Center Institutional Animal Care & Use Committee. Mice were anesthetized by isoflurane inhalation (2–3%) during implantation and removal of Matrigel plugs, and euthanized by cervical dislocation.

### Cell culture

Primary human dermal lymphatic endothelial cells (LECs) isolated from adult skin were purchased from PromoCell GmbH (Heidelberg, Germany, Lot # 478Z013.2) and cultured in endothelial cell growth medium MV2 (PromoCell) supplemented with endothelial growth bullet kit along with 5% fetal bovine serum (FBS) and 1% penicillin (100 IU/mL)-streptomycin (100 ug/mL) solution. LEC culture was maintained at 37°C with 5% CO_2_ in a humidified incubator, and cells were used till passage 8 for experiments. In order to validate the identity of these cells, we performed quantitative real-time PCR (qRT-PCR) experiments to determine the mRNA expression of key LEC markers, *FLT4 (VEGFR3)*, *LYVE-1* and *podoplanin* (*PDPN*) in these LECs and compared them with human aortic endothelial cells (HAoEC, Lonza) (**S1 Table**). The qRT-PCR data demonstrated significantly higher mRNA expression of all these markers in LECs compared with HAoEC **(S1 Fig)**.

### Western blot

SDS-PAGE and Western blotting were performed according to standard procedures [15]. Protein concentrations in cell lysates were quantified using Pierce BCA Protein Assay Kit (Thermo Scientific). Equal amounts of proteins were separated using SDS-PAGE gels, transferred onto nitrocellulose membranes (Li-Cor Biosciences), and membranes were blocked with intercept blocking buffer. Blocked membranes were probed with the following primary antibodies: phospho-eNOS (Ser1177), total eNOS, phospho-AKT (Ser473), total AKT, phospho-ERK1/2 (Thr202/Tyr204), total ERK1/2 and GAPDH. Membranes probed with primary antibodies were incubated with IRDye-conjugated secondary antibodies (Li-Cor Biosciences), washed, and scanned using an Odyssey DLx Infrared Imaging System. Quantification of band intensities was carried out using the NIH ImageJ software.

### Quantitative real-time PCR

Total RNA was extracted from LECs using TRIzol Reagent (Ambion) according to the manufacturer’s protocol. RevertAid RT Kit (Thermo Scientific) was used to reverse transcribe RNA (500 ng) into complementary DNA. The qRT-PCR reactions were performed in a QuantStudio 3 Real-Time PCR System (Applied Biosystems) employing PowerUp SYBR™ Green Master Mix (Thermo Scientific) using gene-specific primers for *FLT4*, *LYVE-1* and *PDPN* (**S1 Table**). GAPDH was taken as a reference gene, and relative gene expression analyzed using the 2-ΔΔCt method.

### LEC proliferation assay

Cell proliferation was determined using Cell Proliferation reagent WST-1. Briefly, cells (10,000 cells/well) seeded in a 96-well culture plate were treated with vehicle (PBS) or NaHS (10, 20, 30, 50 μM) in basal medium MV2 (PromoCell GmbH) containing 0.5% FBS for 48 h. At the end of the incubation period, 10 μL WST-1 reagent was added to each well and incubated at 37°C for 4 h. The absorbance at 450 nm was measured using a Cytation 5 imaging reader (BioTek, Vermont, USA), and absorbance at 690 nm was taken as a reference.

In addition, the number of Ki67-positive nuclei as a marker of proliferation was determined using immunostaining experiments. LECs were plated on coverslips placed in a 24-well culture plate and treated with vehicle or NaHS (30 μM) for 24 h. After 24 h, cells were fixed with 2% paraformaldehyde (PFA), permeabilized with 0.1% triton X-100, blocked, and incubated with Ki67 primary antibody (1:100) overnight at 4°C. Then, coverslips with cells were washed and incubated with Alexa Flour 594-labelled secondary antibody (1:500) and Alexa Fluor 488-phalloidin at room temperature. Coverslips were washed and mounted on slides with a DAPI-containing mounting media. Images of six randomly selected microscopic fields were taken using a Zeiss 710 confocal microscope, and the number of Ki67-positive nuclei was counted using the NIH ImageJ software.

### LEC migration assay

LEC migration in response to NaHS treatment was investigated using transwell migration assay (Boyden Chamber, 8.0 μm pore polycarbonate membranes, Corning, NY, USA). Cells (20,000 cells/insert) were seeded on transwell inserts in basal medium MV2 (0.5% FBS) with/without NaHS (30 μM), and the lower chamber was filled with complete endothelial cell growth medium MV2. Cells were incubated at 37° C to allow cell migration for 24 h. Non-migrated cells from the topside of membranes were wiped, and membranes were fixed with 4% PFA and stained with 0.5% crystal violet. Images of at least seven randomly selected microscopic fields were acquired using an inverted phase-contrast microscope, and the number of cells migrated to the lower side of the membrane determined using the NIH ImageJ software.

### LEC tube formation assay

Formation of tube networks by LECs was assessed as described earlier [16, 17]. Growth factor-reduced Matrigel solution was used to coat wells of a 96-well culture plate for 45 min at 37°C. LECs (20,000 cells/well) in basal medium MV2 media (0.5% FBS) with or without NaHS (30 μM) were seeded onto solidified Matrigel and incubated for 6 h and 14 h. After fixation with 4% PFA, images of tube networks were captured using an inverted phase-contrast microscope. Tube length, number of branching points and loops were determined using the NIH ImageJ software.

### Intracellular NO analysis

LECs seeded at a density of 20,000 cells/well in a 96-well culture plate, were treated with vehicle or NaHS (30 μM) for 24 h. At the end of incubation period, cells were washed and incubated with DAF-FM diacetate solution (5 μM) in serum-free media for 45 min at 37°C. Then, cells were washed with sterile PBS and incubated in serum-free basal media MV2 for 30 min, and fluorescence intensity was measured using excitation/emission spectra 495/515 nm with a Cytation 5 imaging reader.

### Reactive oxygen species generation

The effect of NaHS treatment on intracellular reactive oxygen species (ROS) production was determined using 2′,7′-dichlorodihydrofluorescein diacetate (H2DCFDA) [18]. LECs seeded in a 6-well culture plate were treated with NaHS (30 μM) for 1 h. Cells were then washed with sterile PBS and incubated with H2DCFDA (5 μM) for 30 min at 37°C. Flow cytometry analysis (Ex: 492 nm, Em: 525 nm) was performed using an Agilent NovoCyte flow cytometer, and mean fluorescence intensities were compared among groups.

### Cell cycle analysis

LECs seeded in a 12-well culture plate were pretreated with NaHS (100 μM) for 1 h (or as indicated) and incubated with oxLDL (100 μg/mL) for 24 h. After 24 h, cells were washed with sterile PBS and fixed with ice-cold 70% ethanol for 2 h at 4°C. Then, cells were stained with FxCycle™ PI/RNase Staining Solution for 30 min at room temperature in dark. The samples were analyzed using an Agilent NovoCyte flow cytometer.

### In vivo lymphatic vessel formation assay

In vivo lymphatic vessel formation was determined using the Matrigel plug assay. Ten-week-old male C57BL/6 wild-type mice were anesthetized by isoflurane inhalation (2–3%) and level of anesthesia was determined by toe pinch. The anesthetized mice were injected subcutaneously (flank region) with 400 μL growth factor-reduced Matrigel premixed with PBS or NaHS (30 μM) using an ice-cold syringe, and Matrigel was allowed to polymerize for 2 min before returning mice to their respective cages. After 10 days of Matrigel plug implantation, mice were anesthetized by isoflurane inhalation and euthanized by cervical dislocation. Matrigel plugs from these mice were harvested, fixed in 4% PFA, embedded in paraffin blocks and sectioned. Paraffin sections were deparaffinized, rehydrated and underwent antigen retrieval (citrate buffer at 98°C for 10 min). Next, sections were blocked and incubated with anti-LYVE-1 (Abcam; ab14917, 1:100) and CD31 (R&D Systems, AF3628, 1:100) antibodies to determine lymphatic and blood vessel density respectively. In addition, to investigate immune cell infiltration in Matrigel plugs, sections were incubated with CD45 (Cell Signaling Technology; 70257S, 1:70) antibody overnight at 4°C. Next, sections were washed, incubated with fluorophore-conjugated secondary antibodies for 1 h at room temperature, followed by mounting with a DAPI-containing Fluoromount-G (Thermo Fisher Scientific). Fluorescent images were captured using a Zeiss 710 confocal microscope. To determine macrophage accumulation in Matrigel plugs, sections were immunostained with CD68 (Thermo Fisher Scientific, MA5-13324, 1:100) antibody and detected using Epredia UltraVision LP HRP polymer and DAB Detection System (Richard Allan Scientific LLC) according to the manufacturer’s instructions. Images were acquired using an Olympus BX43 inverted microscope. For staining experiments, three sections from each Matrigel plug were utilized and images of five random microscopic fields were captured. Hematoxylin and eosin staining was conducted using the standard protocol. All images were analyzed using the Image-Pro Plus software (Media Cybernetics, Bethesda, MD).

### Statistical analysis

Data are presented as mean ± SEM. Statistical tests were performed with GraphPad Prism 9 (La Jolla, CA, USA). The sample number (*n*) for each experiment/group is mentioned in the figure legends. The normality of data was determined using Shapiro-Wilk normality test. Student’s *t* test was employed for comparison between two groups (parametric data), while one-way or two-way ANOVA with post hoc tests whichever appropriate, was utilized for comparing multiple groups. A *p*-value less than 0.05 was considered statistically significant.

## Results

### NaHS treatment enhances LEC proliferation and tube formation in vitro

**Fig 1:**
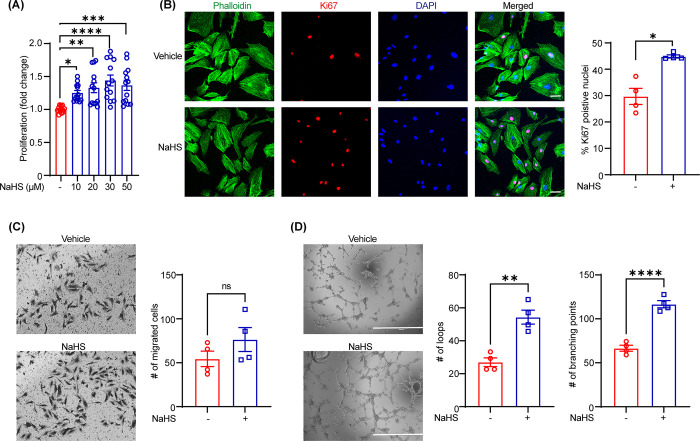
NaHS treatment enhances LEC proliferation and tube formation in vitro. **(A)** Human dermal lymphatic endothelial cells (LECs) were treated with PBS (vehicle) or various concentrations of NaHS (10–50 μM) for 48 h and cell proliferation determined using WST-1 assay. The data are representative of four independent experiments conducted at least in duplicate. **(B)** LECs plated on coverslips were treated with or without NaHS (30 μM) for 24 hours. Cells were immunostained for Ki67 (red) and nuclei counterstained with DAPI (blue). Microscopic images of five random fields were captured. Representative images are shown. Scale bar 50 μm. The bar graph shows the percentage of Ki67-positive nuclei (*n* = 4). **(C)** Representative images of LEC migration after treatment with vehicle or NaHS (30 μM, 24h). The bar diagram shows the number of migrated cells (*n* = 4). **(D)** LECs were seeded in wells of a Matrigel-coated-96-well plate in basal media (0.5% FBS) containing vehicle or NaHS (30 μM), and tube formation was investigated after 6 h. Representative images of tube formation are shown. Scale bar 1000 μm. Images of 3 random fields were captured, and the number of loops and branching points was quantified (*n* = 4). Statistical analyses were performed using one-way ANOVA with Dunnett multiple comparisons test **(A)**, and two-tailed unpaired student *t* test **(B-D)**. Data represent mean ± SEM. *p < 0.05, **p < 0.01, and ****p < 0.0001.

To determine the effects of exogenous NaHS treatment on lymphatic vessel formation, we first investigated LEC proliferation following incubation with different concentrations of NaHS (0–50 μM) using WST-1 assay. As shown in **Fig 1A**, NaHS treatment significantly enhanced the proliferation of LECs at all studied concentrations, however, cells treated with 30 μM NaHS concentration demonstrated maximum proliferation. Therefore, we selected 30 μM NaHS concentration for further experiments. Next, we performed immunostaining experiments to determine the number of Ki67-positive LECs in response to NaHS treatment. Immunofluorescence data revealed an increased number of Ki67-positive nuclei in NaHS treatment group compared with vehicle-treated control cells (**Fig 1B**) confirming the proliferative effect of NaHS on LECs.

The migration and tube-forming ability of LECs are crucial for the formation of lymphatic vessels [19]. Hence, we studied the effects of NaHS treatment on LEC migration and tube formation. NaHS treatment did not stimulate LEC migration as assessed using transwell migration assay (**Fig 1C**). Further, we performed the Matrigel-based tube formation assay to investigate whether NaHS promotes LEC tube formation. Consistent with proliferative effects of NaHS on LECs, NaHS treatment augmented LEC tube formation as demonstrated by increased number of loops, branching points and tube length in comparison to control cells at both shorter (6 h) and longer time points (14 h) (**Fig 1D and S2 Fig**). Collectively, these findings suggest the pro-lymphangiogenic effects of NaHS treatment in vitro.

### NaHS treatment stimulates pro-angiogenic AKT-eNOS signaling in LECs

**Fig 2:**
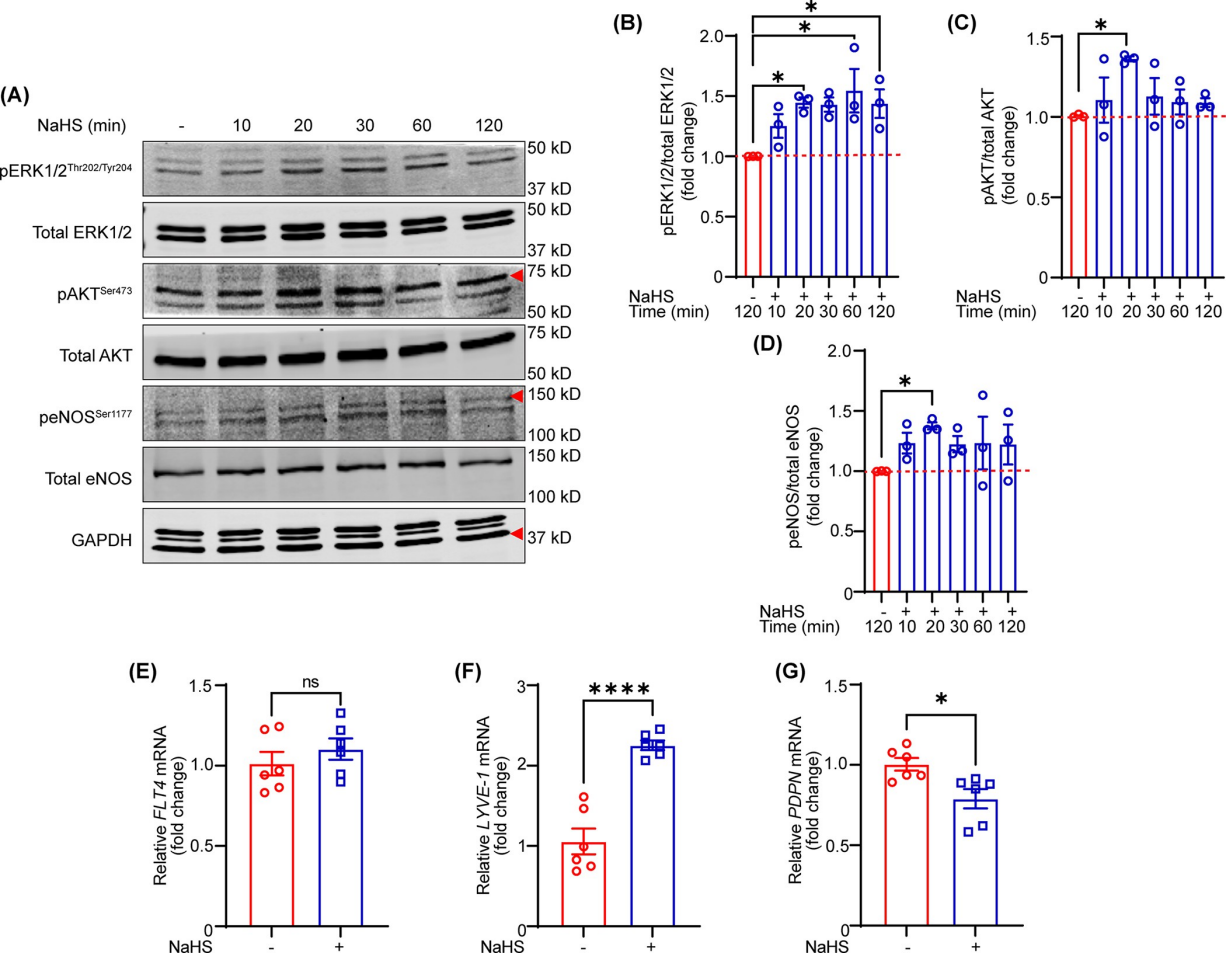
NaHS treatment stimulates pro-angiogenic AKT-eNOS signaling in LEC. **(A-D)** LECs were serum-starved overnight (16 h) in basal media containing 0.5% FBS and treated with NaHS (30 μM) in basal media (0.5% FBS) for the indicated time intervals (10–120 min). Cell lysates were subjected to western blot analysis. Representative Western blot images are shown. Bar diagrams illustrate the ratios of phospho to total proteins (*n* = 3). **(E-G)** LECs were treated with or without NaHS (30 μM) in basal media (0.5% FBS) for 24 h to investigate *FLT4*, *LYVE-1* and *PDPN* transcripts expression using qRT-PCR (*n* = 6). Statistical analyses were performed using one-way ANOVA with Sidak **(B)** and Dunnett multiple comparisons test **(C and D)** and two-tailed unpaired student *t* test **(E-G)**. Data represent mean ± SEM. *p < 0.05 and ****p < 0.0001.

Activation of PI3K-AKT-eNOS and ERK1/2 is the well-studied pro-angiogenic signaling and plays a pivotal role in lymphangiogenesis [20–22]. To investigate the signaling pathways by which NaHS stimulates lymphatic vessel formation, LECs were treated with/without NaHS (30 μM) for different timepoints (10–120 min), and cell lysates were subjected to western blot experiments. The immunoblotting data demonstrated increased phosphorylation of ERK1/2 (Thr202/Tyr204), AKT (Ser473) and eNOS (Ser1177) after 20 min of incubation with NaHS (**Fig 2A–2D**). These results indicate that NaHS-stimulated AKT-eNOS signaling may be responsible for increased lymphatic vessel formation.

To determine the effects of NaHS treatment on mRNA expression of LEC-specific genes including *FLT4*, *LYVE-1* and PDPN, we treated LECs with vehicle or NaHS (30 μM) for 24 h and performed qRT-PCR experiments. The qPCR data showed increased mRNA expression of *LYVE-1* and reduced levels of *PDPN* mRNA in NaHS-treated cells in comparison to control cells, however, no differences in *FLT4* transcript levels were observed between the both groups **(Fig 2E–2G).**

### NaHS supplementation promotes NO production, suppresses ROS generation and stimulates cell cycle progression in LECs

**Fig 3:**
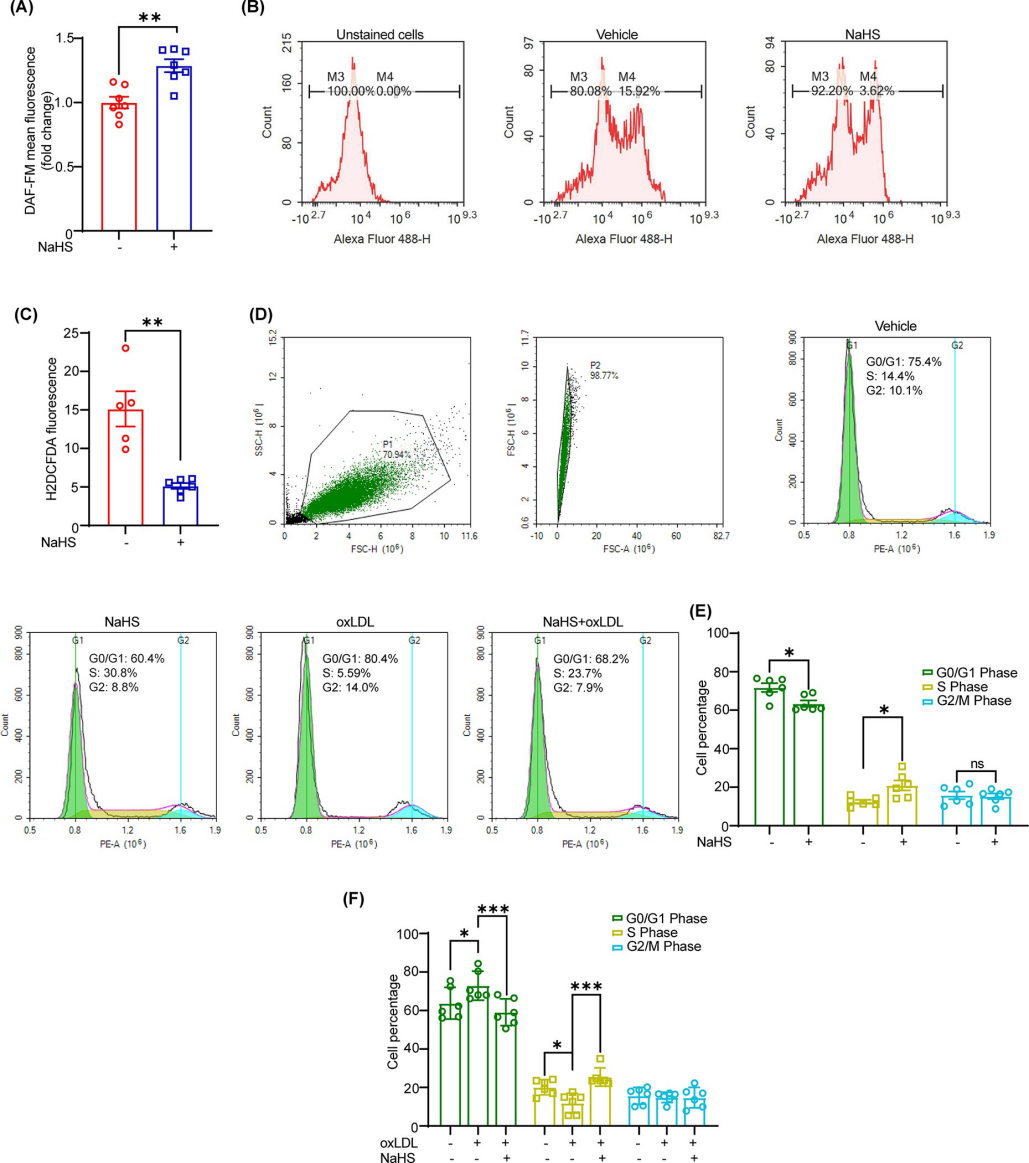
NaHS promotes NO production, inhibits ROS generation, and stimulates cell cycle progression in LECs. **(A)** LECs were treated with or without NaHS for 24 h and intracellular NO generation quantified using DAF-FM diacetate solution (*n* = 7). **(B and C)** LECs were treated with or without NaHS for 1 h and incubated with H2DCFDA solution to measure intracellular ROS production using flow cytometry. Representative histograms are shown. The bar graph represents the mean percentage of cells with H2DCFDA fluorescence in both groups (*n* = 5). **(D-E)** Vehicle- or NaHS-treated cells (30 μM, 24h) were analyzed for cell cycle progression. Representative histograms are shown. The bar graph shows the percentage of cells in various phases of cell cycle (*n* = 6). **(F)** Cell cycle analysis to determine the effects of NaHS pretreatment on oxLDL-induced inhibition of cell cycle in LECs. Bar graphs show the cell percentage in various cell cycle phases (*n* = 6). Statistical analyses were performed using a two-tailed unpaired student *t* test **(A and C)** and two-way ANOVA with Tukey multiple comparisons test **(E and F)**. Data represent mean ± SEM. *p < 0.05, **p < 0.01, and ***p < 0.001.

Since NO is required for lymphangiogenesis [23] and NaHS treatment enhances eNOS activation in LECs (**Fig 2A**), which is the major source of NO production in these cells [16], therefore, we examined NO production following NaHS treatment. As shown in **Fig 3A**, NaHS-exposed cells have higher production of NO compared with vehicle-treated cells. It is known that increased generation of reactive oxygen species (ROS) superoxide anion (O_2_^•-^) scavenges NO and reduces NO bioavailability for lymphangiogenesis [16, 18]. Hence, we next investigated the effects of NaHS treatment on ROS production in LECs. Our flow cytometry data quantifying H2DCFDA fluorescence as a measure of ROS generation showed that NaHS treatment reduces ROS generation in LECs (**Fig 3B and 3C**). Besides, cell cycle analysis demonstrated an increased percentage of cells in the S phase of cell cycle and significantly decreased cell percentage in the G0/G1 phase in NaHS-treated group compared with vehicle, however, no significant differences were observed in the percentage of cells in the G2/M phase (**Fig 3D and 3E**). Taken together, these data suggest that NaHS reduces ROS generation, promotes NO production and enhances cell cycle progression in LECs.

### Treatment with NaHS abrogates the inhibitory effects of oxLDL on cell cycle

It has been demonstrated that oxLDL inhibits lymphatic vessel formation and causes cell cycle arrest in LECs [19, 24]. Therefore, we investigated the effects of NaHS treatment on oxLDL-induced inhibition of cell cycle. Interestingly, our results demonstrated that NaHS treatment suppresses the inhibitory effects of oxLDL on LEC cell cycle as shown by improved percentage of cells in the S phase and reduced frequency of cells in the G0/G1 phase following NaHS+oxLDL treatment compared with oxLDL-treated cells (**Fig 3D and 3F**). These findings indicate that pretreatment with NaHS prevents oxLDL-induced cell cycle arrest in LECs, and NaHS may be used to alleviate the anti-lymphangiogenic effects of oxLDL.

### NaHS promotes in vivo lymphatic vessel formation

**Fig 4:**
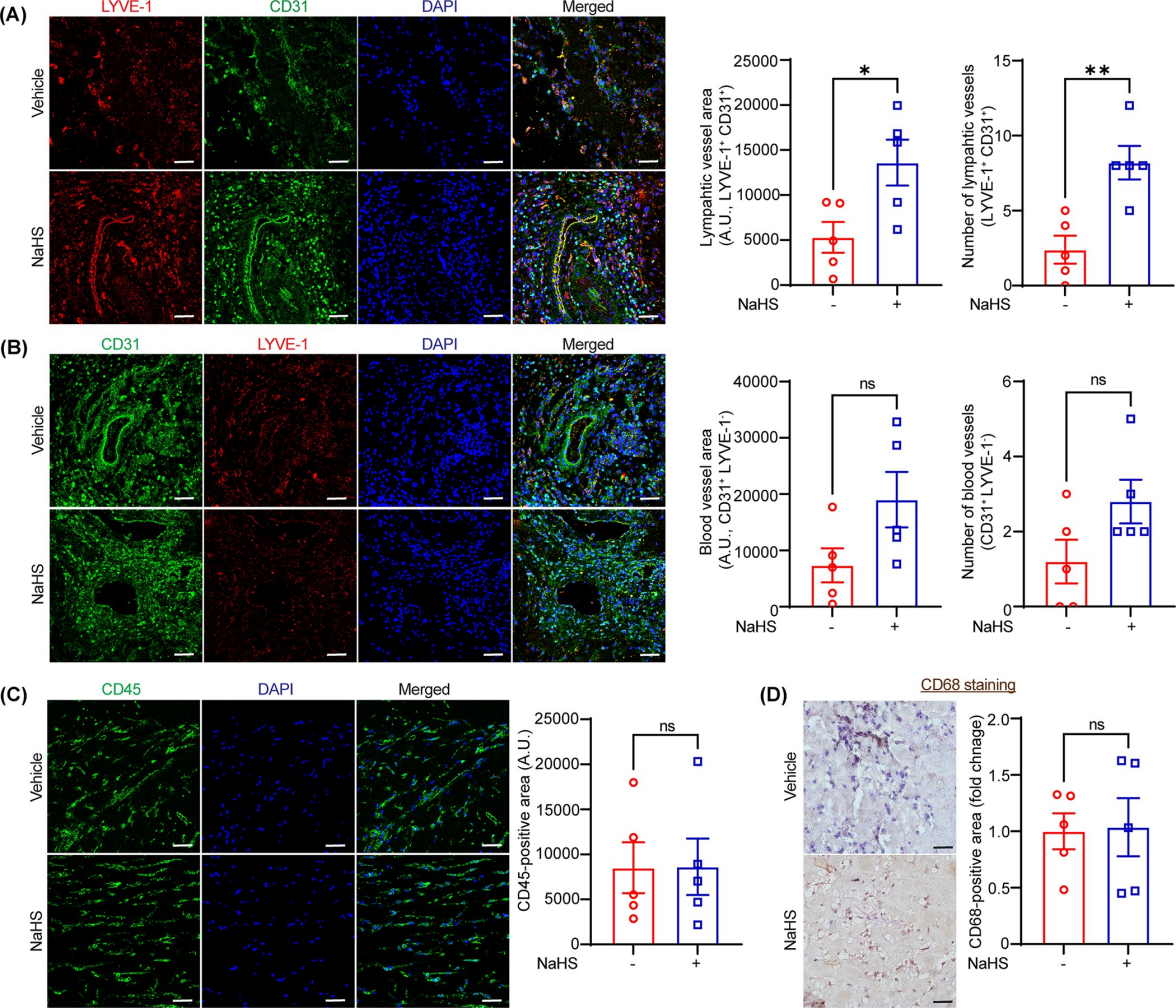
NaHS promotes in vivo lymphatic vessel formation. **(A-C)** Wild-type male mice were injected subcutaneously with 400 μL of Matrigel solutions premixed with either PBS or NaHS (30 μM). Matrigel plugs were isolated after 10 days of implantation, embedded in paraffin blocks and paraffin sections were double immunostained for LYVE-1 **(red, A)**, CD31 **(green, B)**, and CD45 **(green, C)**, CD68 **(macrophage, D)**. Representative images of immunostaining are shown. CD31^+^ LYVE-1^+^ vessel-like structures: lymphatic vessels and CD31^+^ LYVE-1^-^ structures: blood vessels. Scale bar 50 μm. Statistical analyses were done using a two-tailed unpaired student *t* test. Data represent mean ± SEM. *p < 0.05.

To determine the role of exogenous NaHS administration on in vivo lymphatic vessel formation, the Matrigel plug experiments were performed. Male wild-type mice were implanted subcutaneously with Matrigel premixed with PBS or NaHS (30 μM). Matrigel plugs were harvested (10 days) and their paraffin cross-sections were immunostained with LYVE-1 (lymphatics), CD31 (blood/lymphatic vessels) and CD45 (immune cells), CD68 (macrophages) antibodies to determine LV and blood vessel density, immune cell accumulation and macrophage accumulation [15]. We referred CD31^+^ LYVE-1^+^ vessel-like structures as lymphatic vessels, while CD31^+^ LYVE-1^-^ structures as blood vessels. Interestingly, immunostaining results of Matrigel plug sections demonstrated increased lymphatic vessel area and number of lymphatic vessels in plugs containing NaHS compared with PBS-containing plugs (**Fig 4A and S3 Fig**). However, there were no significant differences in blood vessel area and number of blood vessels between both groups (**Fig 4B**). Additionally, no differences were found in CD45- and CD68-positive areas between both groups (**Fig 4C and 4D**). Collectively, these results suggest that NaHS treatment induces LV formation in vivo without affecting accumulation of immune cells.

## Discussion

The lymphatic vasculature is important for the maintenance of tissue homeostasis and resolution of inflammation via draining extravasated fluid, immune cells, hormones, lipids and cytokines. Several studies have shown that reduced LV density and decreased lymphatic clearance lead to the development of pathological diseases including atherosclerosis, hypertension, diabetes, obesity, chronic lung disease, renal fibrosis, and edema, and improved lymphatic function alleviates these pathological conditions [14, 25–27]. In the present study, we investigated the effects of NaHS (H_2_S donor) treatment on lymphatic vessel formation using in vivo and in vitro approaches and report its pro-lymphangiogenic potential and underlying mechanisms.

H_2_S is a gasotransmitter found in minute quantity in human body and produced enzymatically by cystathionine-γ-lyase (CSE), cystathionine-β-synthase and cysteine aminotransferase/3-mercaptosulfurtransferase [28]. It serves as a signaling molecule and confers protective effects in cardiovascular disease due to its pro-angiogenic, antioxidant, antiinflammatory and vasodilatory functions [29–32]. The role of H_2_S in physiological and pathological angiogenesis has been widely studied [33]. However, our knowledge of the ability of H_2_S in regulating lymphangiogenesis and underlying molecular mechanisms is limited. To investigate the role of H_2_S in modulating lymphatic vessel formation, we utilized NaHS, a well-known fast-releasing H_2_S donor and determined its effects on LEC proliferation, migration and tube formation. It is known that NaHS rapidly releases H_2_S and show its effects within seconds. It has been widely utilized in both in vitro and in vivo studies, while other H_2_S donors like 5-(4-hydroxyphenyl)-1,2-dithiol-3-thione release H_2_S enzymatically and are slow in inducing molecular signaling. Our in vitro data showed improved lymphatic vessel formation (cell proliferation and tube formation) by LECs treated with NaHS. These results are consistent with Suzuki et al. who showed improved LECs’ tube-forming capacity following treatment with an H2S donor diallyl trisulfide (DATS) [14]. H_2_S activates vascular endothelial growth factor receptor (VEGFR) 2 in vascular endothelial cells and promotes downstream activation of AKT and ERK [33, 34]. Further, H_2_S can increase eNOS phosphorylation at its activating site S1177 via PI3K-AKT-mediated signaling in endothelial cells [35]. Treatment with H_2_S donor augments phosphorylation of eNOS in myocardial tissue of CSE null mice suggesting its role in enhancing NO production and signaling [36]. Similar to vascular endothelial cells, our Western blot data demonstrated increased activation of ERK1/2, AKT and eNOS in NaHS-treated LECs compared with control cells. A recent study also suggested induced AKT phosphorylation in LECs with DATS treatment, however, the authors did not study the effects of DATS on eNOS activation [14]. VEGF-C-induced VEGFR3 activation is the major signaling mechanism that stimulate lymphangiogenesis [18, 37, 38]. Our qRT-PCR data demonstrated increased mRNA expression of *LYVE-1* in NaHS-treated LECs compared with control cells with no changes in *FLT4* transcript levels, suggesting a potential role LYVE-1-mediated signaling in promoting LEC proliferation and migration, which may subsequently facilitate lymphatic vessel formation.

H_2_S and NO has been shown to interact and regulate the production of each other in cardiovascular system [35, 39], and NO is an important lymphangiogenesis stimulator [23]. Additional experiments performed to determine the effects of NaHS treatment on NO generation showed improved intracellular NO production by NaHS-exposed LECs, indicating the role of H_2_S in enhancing NO concentration in these cells. Increased amount of ROS can reduce NO bioavailability and inhibit lymphatic vascularization [18, 40]. Consistently, NaHS treatment attenuated ROS production in LECs. However, we believe that scavenging of ROS with antioxidants may not have similar pro-lymphangiogenic effects as of NaHS treatment since optimal amount of ROS is required for proper functioning of cells. Previous studies have demonstrated that AKT promotes cell cycle progression by inducing the expression of various cyclins and suppressing the levels of cell cycle inhibitors such as p27 and p21 [41, 42]. Consistent with these studies and increased AKT activation with NaHS treatment, our flow cytometry data indicated that H_2_S induces cell cycle progression and rescues the inhibitory effects of oxLDL on cell cycle in LECs [19, 24]. Earlier study by Singla et al. reported that oxLDL inhibits lymphangiogenesis and causes cell cycle arrest in LECs by activating CD36-mediated mechanisms, and CD36 is involved in LDL uptake by various cell types [19]. Recently, Yu and colleagues reported reduced membranous CD36 levels in cardiac tissue of NaHS-treated diabetic rats compared with control rats [43]. Therefore, it is tempting to speculate that NaHS treatment via downregulating cell surface expression of CD36 protein improved cell cycle progression in oxLDL-treated LECs. Consistent with the pro-lymphangiogenic effects of NaHS in vitro, Matrigel plug assay demonstrated increased CD31^+^ LYVE-1^+^ LV area in NaHS-containing plugs compared with vehicle plugs. However, no differences in immune cell/macrophage infiltration and blood vessels area/number were observed. These data hint that NaHS may promote in vivo lymphangiogenesis independent of immune cell-derived pro-lymphangiogenic factors [44].

In conclusion, the present study demonstrates that H_2_S donor NaHS promotes in vitro and in vivo lymphatic vessel formation, induces AKT-eNOS signaling and NO production, and enhances cell cycle progression in LECs, identifying NaHS as a potential therapeutic agent to stimulate lymphangiogenesis.

## Supporting information

S1 Raw images Uncropped western blot images. (PDF)

S1 Fig Characterization of LECs used in the present study. The qRT-PCR data showing mRNA expression of LEC markers, *FLT4*, *LYVE-1* and *PDPN*, in human dermal LECs (HDLEC) and human aortic endothelial cells (HAoEC) (*n* = 6). A two-tailed unpaired student *t* test was performed to analyze significance. Data represent mean ± SEM. **p < 0.01, ****p < 0.001.
(TIF)

S2 Fig NaHS treatment induces LEC tube formation in vitro. LECs were seeded in wells of a Matrigel-coated-96-well plate in basal media (0.5% FBS) containing vehicle or NaHS (30 μM), and tube formation was investigated after 14 h. Representative images of tube formation are shown. Scale bar 1000 μm. Images of 3 random fields were captured, and tube length and branching points were quantified (*n* = 5). Statistical analyses were done by employing a two-tailed unpaired student *t* test. Data represent mean ± SEM. *p < 0.05.
(TIF)

S3 Fig Representative images of hematoxylin and eosin staining of Matrigel plug sections. (TIF)

S1 Table List of primers used for mRNA quantitation using quantitative real-time PCR. (PDF)

## Abbreviations

H_2_S: Hydrogen sulfide
LECs: Lymphatic endothelial cells
LVs: Lymphatic vessels
NaHS: Sodium hydrogen sulfide
eNOS: Endothelial nitric oxide synthase
H2DCFDA: 2′,7′-dichlorodihydrofluorescein diacetate
LYVE-1: Lymphatic vessel endothelial hyaluronan receptor-1
NO: Nitric oxide
ROS: Reactive oxygen species
