# Supplementary figures and images for: Hydrogen sulfide donor activates AKT-eNOS signaling and promotes lymphatic vessel formation
Source: PLoS One. 2023 Oct 26;18(10):e0292663. doi: 10.1371/journal.pone.0292663 (PMC10602273; doi:10.1371/journal.pone.0292663)

Figure 2 A

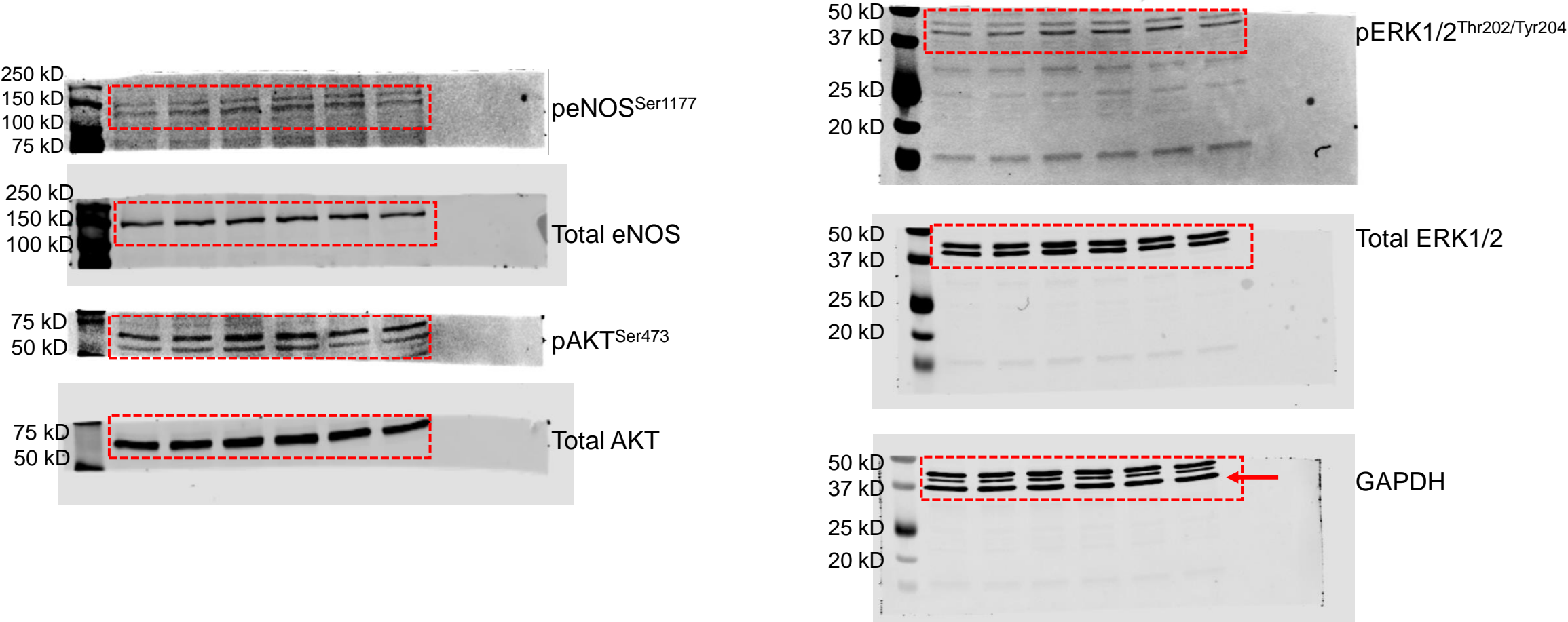

Supplement: S1 Raw images — (PDF) [file pone.0292663.s001.pdf]

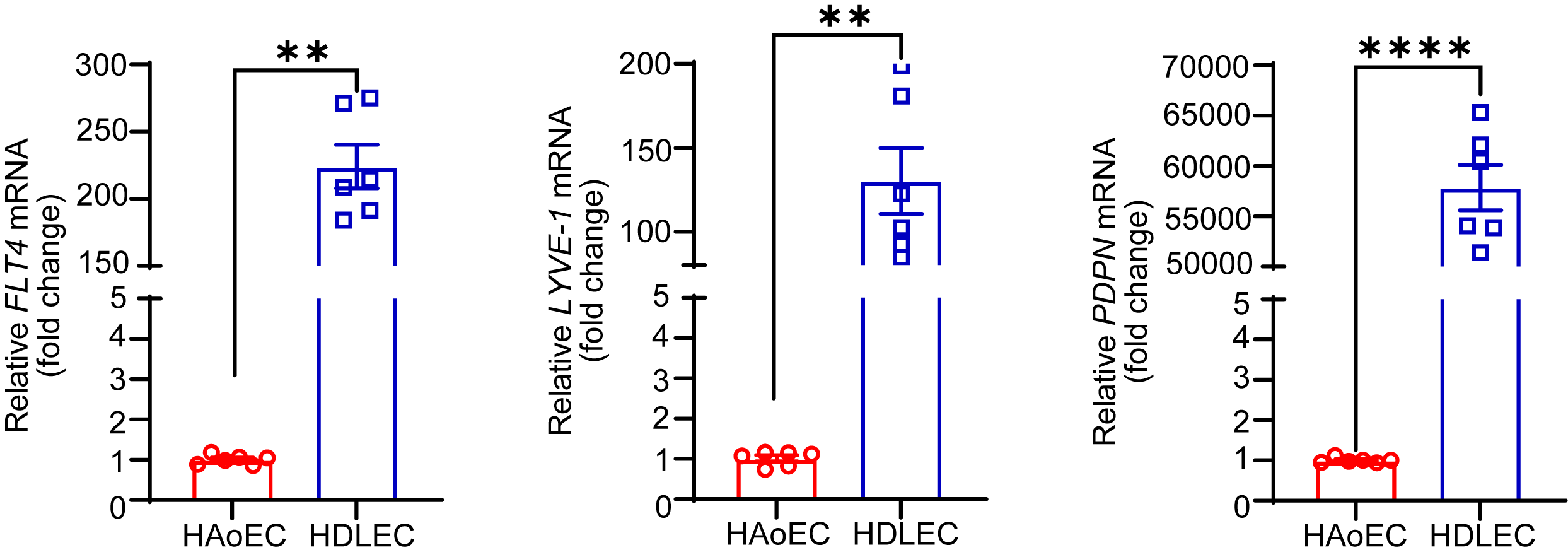

Supplement: S1 Fig — The qRT-PCR data showing mRNA expression of LEC markers, FLT4, LYVE-1 and PDPN, in human dermal LECs (HDLEC) and human aortic endothelial cells (HAoEC) (n = 6). A two-tailed unpaired student t test was performed to analyze significance. Data represent mean ± SEM. **p < 0.01, ****p < 0.001. (TIF) [file pone.0292663.s002.tif]

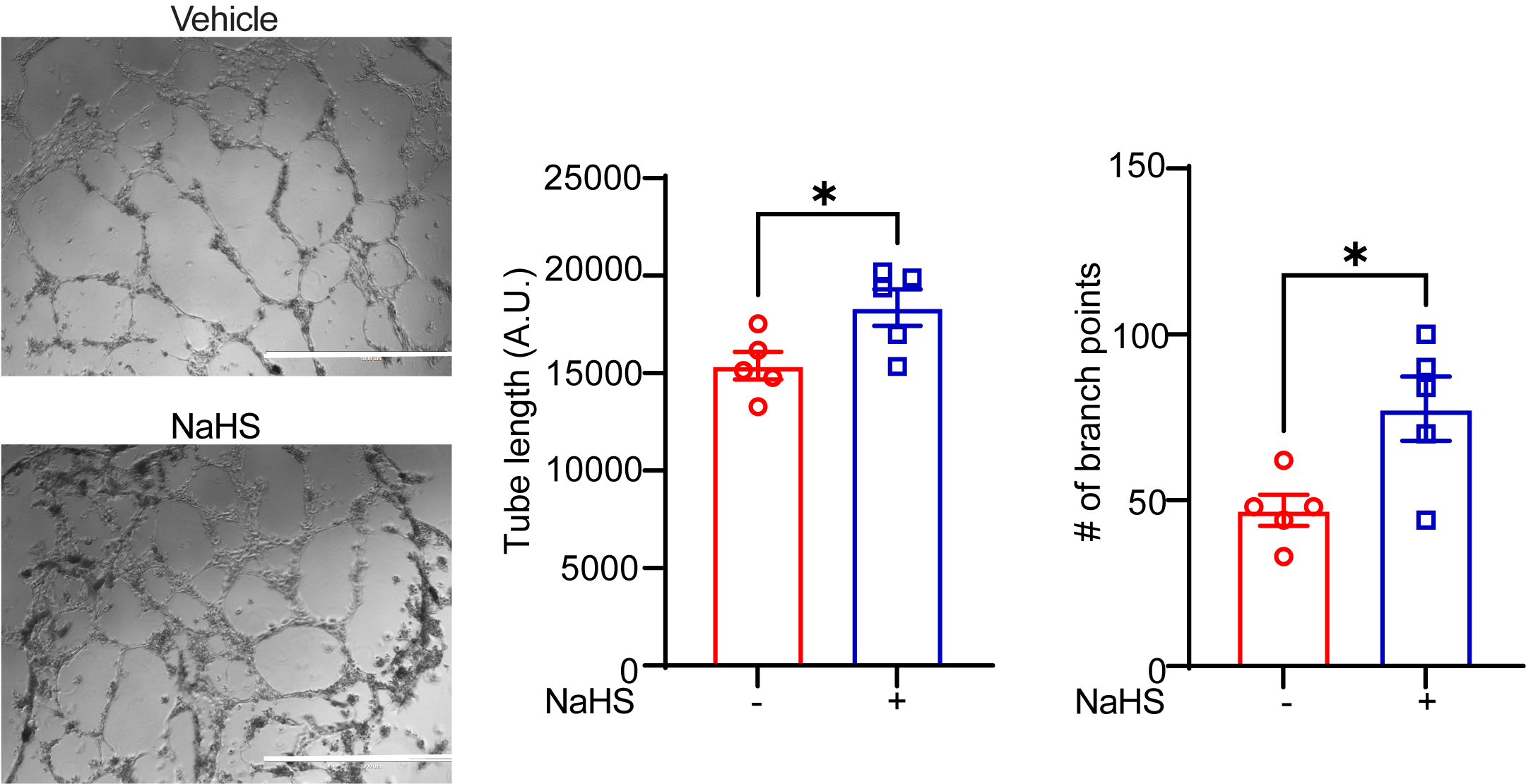

Supplement: S2 Fig — LECs were seeded in wells of a Matrigel-coated-96-well plate in basal media (0.5% FBS) containing vehicle or NaHS (30 μM), and tube formation was investigated after 14 h. Representative images of tube formation are shown. Scale bar 1000 μm. Images of 3 random fields were captured, and tube length and branching points were quantified (n = 5). Statistical analyses were done by employing a two-tailed unpaired student t test. Data represent mean ± SEM. *p < 0.05. (TIF) [file pone.0292663.s003.tif]

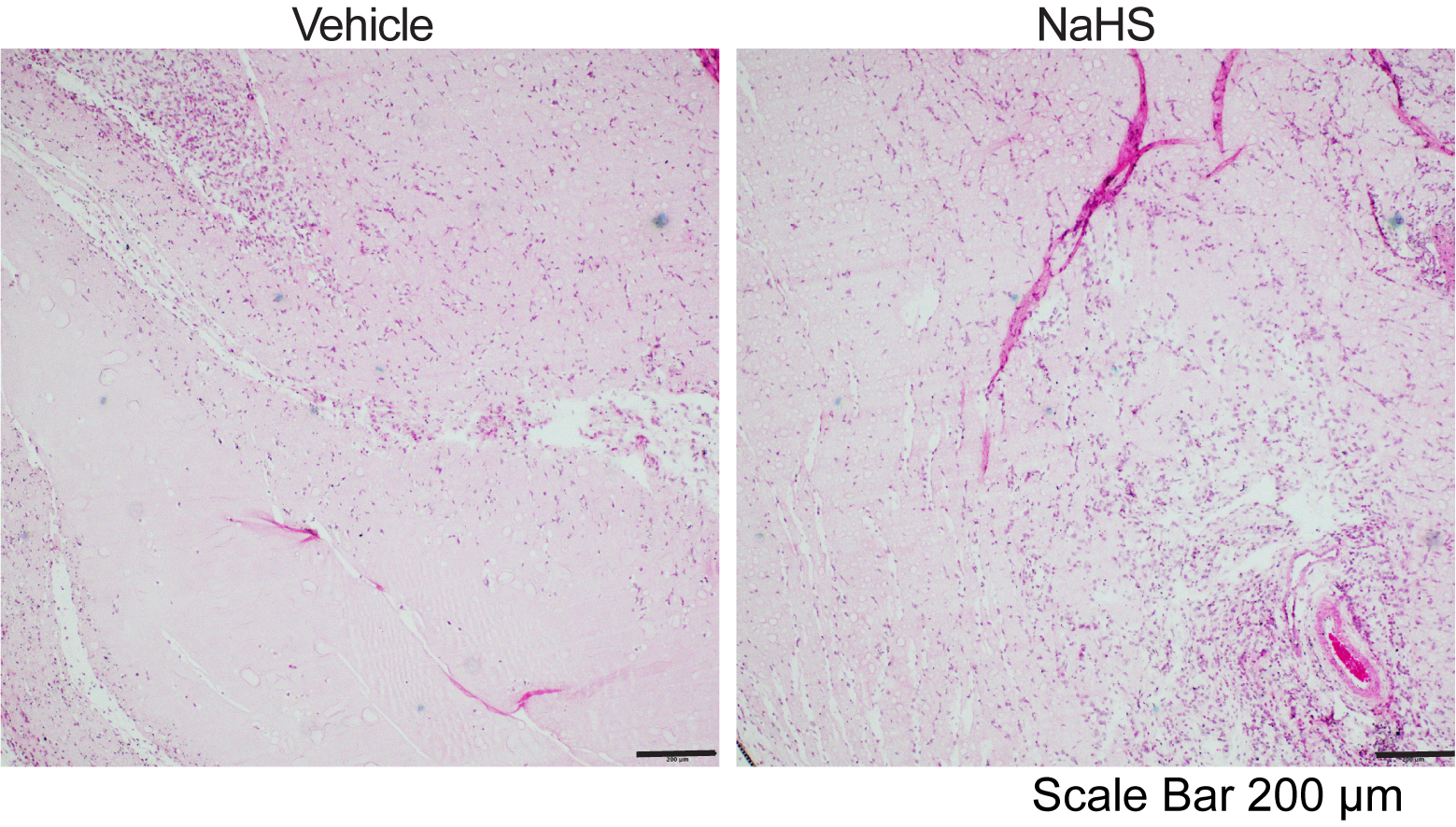

Supplement: S3 Fig — (TIF) [file pone.0292663.s004.tif]
