# Supplementary material for: Hydrogen sulfide donor activates AKT-eNOS signaling and promotes lymphatic vessel formation
Source: PLoS One. 2023 Oct 26;18(10):e0292663. doi: 10.1371/journal.pone.0292663 (PMC10602273; doi:10.1371/journal.pone.0292663)
Supplement: S1 Table — (PDF) [file pone.0292663.s005.pdf]

S1 Table: List of primers used for mRNA quantitation using quantitative real-time PCR.

Gene name	Primer sequences
FLT4	F 5'- CTG GAC CGA GTT TGT GGA GG-3'<br>R 5'- GTC ACA TAG AAG TAG ATG AGC CG-3'
LYVE-1	F 5'- GGG TTG GAG ATG GAT TCG TGG-3'<br>R 5'- ATA GGC TGC AAA CTG TCG GC-3'
PDPN	F 5'- AAC CAG CGA AGA CCG CTA TAA-3'<br>R 5'- CGA ATG CCT GTT ACA CTG TTG A-3'
GAPDH	F 5'- CAT GTT CGT CAT GGG TGT GAA CCA-3'<br>R 5'- AGT GAT GGC ATG GAC TGT GGT CAT-3'
